# Hypothyroidism After Use of Immune Checkpoint Inhibitor Therapy in Patient With Graves' Disease: Cure?

**DOI:** 10.1210/jcemcr/luac024

**Published:** 2022-12-03

**Authors:** Rajeev Sharma

**Affiliations:** Division of Endocrinology, Roswell Park Comprehensive Cancer Center & Jacobs School of Medicine and Biomedical Sciences, University at Buffalo, New York, NY 14203, USA

**Keywords:** immune checkpoint inhibitors, thyroiditis, Graves' disease, hypothyroidism, thyroid-stimulating hormone, programmed cell death protein-1 (PD-1) inhibitor, antithyroid drug (ATD)

## Abstract

Immune checkpoint inhibitors (ICIs) are frequently used as treatment for many malignancies. Immune-related adverse events (irAEs) due to use of ICIs are common. Thyroid involvement is the most common endocrine irAE. Here, we present an unusual case of Graves' disease potentially cured due to destructive thyroiditis caused by inflammation due to ICIs. Thyroid irAEs are more common with programmed cell death protein-1 (PD-1) inhibitor or programmed cell death-ligand 1 (PD-L1) inhibitors than cytotoxic T-lymphocyte-associated protein 4 (CTLA-4) inhibitors. Baseline and serial monitoring of thyroid function tests is recommended.

Immune checkpoint inhibitors (ICIs) are commonly used for treatment of many cancers [1]. The thyroid gland is the most commonly involved endocrine organ in ICI-related toxicities. The majority of patients initially develop thyroiditis followed by permanent hypothyroidism. Very few patients recover with normalization of thyroid function tests. Graves' disease induced by ICIs is quite rare. We report a case of pre-existing Graves' disease that was potentially cured of hyperthyroidism after starting ICIs.

## Case Presentation

**Figure 1.**
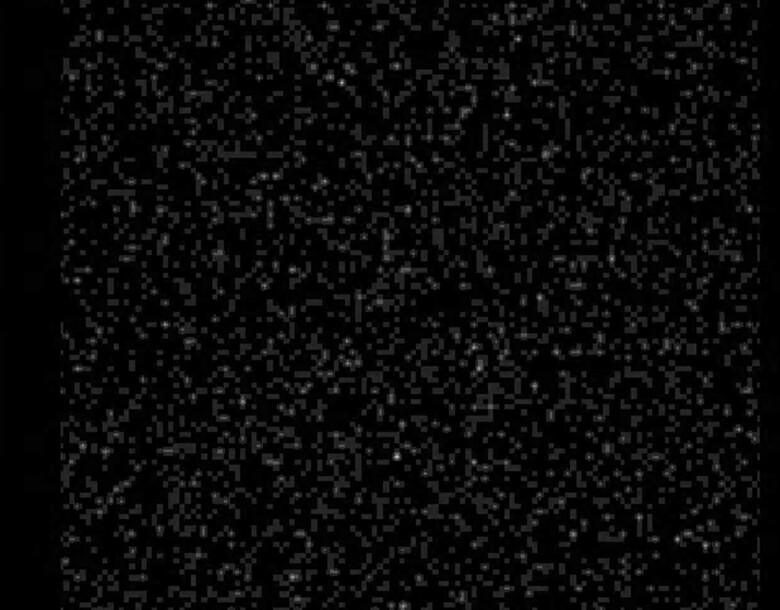
24-hour radioiodine uptake scan showing almost no uptake in thyroid gland.

A 66-year-old man with metastatic lung cancer was referred to the endocrine clinic for suppressed thyroid-stimulating hormone (TSH) of <0.01 uIU/mL. He was first found to have low TSH about 3 years earlier. At that time, investigations showed suppressed TSH (0.02 uIU/mL), positive thyroid peroxidase (TPO) antibodies, heterogeneous thyroid with no nodules on sonogram and normal 24-hour uptake (12%) on radioactive scan without hot or cold nodules. Antithyroid drugs (ATD) were not prescribed as he was asymptomatic. The patient was subsequently diagnosed with metastatic poorly differentiated squamous cell carcinoma of lung and received chemotherapy with carboplatin and paclitaxel for 4 months. Because of inadequate response, he was switched to the immune checkpoint inhibitor (ICI) pembrolizumab (a programmed cell death protein-1 [PD-1] inhibitor). Pembrolizumab was stopped after 8 cycles due to immune-mediated hepatitis that was treated with prednisone and mycophenolate mofetil. He was not re-challenged with pembrolizumab again. Thyroid function tests prior to starting pembrolizumab were consistent with primary hyperthyroidism (TSH < 0.004 uIU/mL). He was also started on the beta blocker Atenolol by the oncology team for hypertension and low TSH. During initial evaluation in the endocrinology clinic (after the fourth cycle of pembrolizumab), he was diagnosed with autoimmune thyroid disease, likely Graves' disease based on suppressed TSH for the past 3 years, positive TPO antibodies, and family history of Graves' disease in his mother. Thyroid-stimulating immunoglobulin (TSI) came back positive at 1.84 (normal 0 to 0.55 IU/L) confirming the suspicion of Graves' disease. Atenolol was continued and thyroid function tests were followed closely. TSH was found be significantly elevated prior to the seventh cycle of pembrolizumab (Table 1). At that time, the patient was asymptomatic except for fatigue. Thyroid uptake scan at this time showed decreased 24-hour uptake (0.8%) (Fig. 1). A diagnosis of primary hypothyroidism due to ICI therapy was made. He was started on hormone replacement with levothyroxine 50 mcg daily.

**Table 1.** Details of thyroid function tests, antibodies, and levothyroxine dose

Date (months)	TSH (0.4-5 uIU/mL)	FT4 (0.82-1.77 ng/dL)	TT4 (4.9-11.7 ug/dL)	TT3 (53-165 ng/dL)	TSI (0-0.55 IU/L)	TPO (0-34 IU/mL)	Levothyroxine dose (mcg)
0	<0.004(L)	—	12.9 (H)	147 (N)	—	—	None, start of ICI
1	<0.004(L)	—	23 (H)	360 (H)	—	—	None
5	104 (H)	—	<1 (L)	<25 (L)	**Positive**	—	Start 50 mcg/d
6	40 (H)	—	6 (N)	35 (L)	—	—	Increase to 75 mcg/d
8	19 (H)	1.05	—	—	—	—	Increase to 100 mcg/d
11	8.91 (H)	1.24	—	—	—	—	Increase to 112 mcg/d
16	4.26	1.67	—	—	—	—	112 mcg/d
19	0.98	—	10	39 (L)	—	—	112 mcg/d, ICI stopped
24	0.596	1.68	—	—	—	—	Decrease to 100 mcg/d
28	0.64	0.9	—	—	—	—	100 mcg/d
34	2.37	1.65	—	—	—	—	100 mcg/d
41	1.82	1.6	—	—	**Negative**	**Negative**	100 mcg/d

Abbreviations: FT4, free thyroxine; H, High, ICI, immune checkpoint inhibitor; L, Low; N/A, not available; TPO, thyroid peroxidase antibody; TSH, thyroid-stimulating hormone; TSI, thyroid-stimulating immunoglobulin; TT3, total triiodothyronine; TT4, total thyroxine.

## Outcome and Follow-Up

Since starting levothyroxine, the dose has been adjusted a few times and patient is doing well. His thyroid antibodies/TSI are now negative at 0.10 (normal 0 to 0.55 IU/L). TSH receptor antibody and thyroid peroxidase antibodies are also negative. Serial thyroid function tests and dates of starting ICI and levothyroxine are detailed in Table 1.

## Discussion

Immune checkpoint inhibitors (ICIs) have changed the paradigm of cancer therapy [1]. Since the approval of ipilimumab (anti CTLA-4 antibody) as the first ICI for treatment of metastatic melanoma in 2011, the use of ICIs has increased many fold [2]. Currently, there are 9 ICIs approved by the Food and Drug Administration (FDA) for the treatment of the cancer. ICIs are monoclonal antibodies that target 2 key signaling pathways related to T-cell activation, namely CTLA-4 and PD-1. Blocking these 2 signaling pathways unleashes T-cell activation leading to destruction of tumor cells. There are broadly 3 classes of ICIs: CTLA-4 inhibitors, PD-1 inhibitors, and PD-L1 inhibitors [3].

ICI therapy is associated with immune-related adverse events (irAEs) with frequent involvement of the endocrine system [4]. Among the endocrine organs, involvement of the thyroid gland is most common, followed by the pituitary gland [5]; ICI-induced type 1 diabetes mellitus is relatively infrequent [6]. Thyroiditis with subsequent progression to primary hypothyroidism is the most common thyroid manifestation [7]. Our patient had initial worsening of hyperthyroidism after starting ICI therapy, which was very likely due to the hyperthyroid phase of an ICI thyroiditis. Some patients can also develop central hypothyroidism due to ICI-related pituitary dysfunction. The incidence of hypothyroidism is estimated to be about 6.6%, whereas hyperthyroidism is reported in about 2.9% with ICI therapy [8]. Graves' disease during ICI therapy is infrequently described in literature. There are a few case reports of ICI-induced Graves' disease [9, 10]. Peiffert and colleagues in a case series of ICI-induced thyrotoxicosis identified 8 patients with Graves' disease at their University Hospital and 8 more patients from a review of the literature [9]. Our patient is an uncommon and probably less reported case of Graves' disease prior to the use of ICI therapy who developed destructive thyroiditis (irAEs) and hypothyroidism. This could be analogous to the use of radioiodine therapy that we use for Graves' disease in noncancer patients. Radioactive iodine scan in our patient showed very low uptake consistent with destructive thyroiditis (Fig. 1). In the case series by Peiffert et al, there were 3 patients with pre-existing Graves' disease who received ICI therapy [9]. One patient was diagnosed on screening, the second patient 1 month later when he was symptomatic, and the third patient was already on ATD treatment prior to ICI therapy. All 3 patients were treated with or continued ATD for various durations (1-23 weeks) along with ICI therapy until hypothyroidism set in. Subsequently all 3 were started on levothyroxine similar to our patient. A total of 6 patients, including the authors’ case series as well as the review of literature, developed spontaneous hypothyroidism over time during the treatment with ICIs. Of the 6 patients who developed hypothyroidism, 4 were also initially treated with ATD. However, our patient, like the other 2/6 patients, did not receive ATD but was only on beta blocker upon presentation. Interestingly, our patient's TSI as well as TPO antibodies became negative after developing ICI-induced thyroiditis and hypothyroidism. One possible explanation for this observation is decreased antibodies formation to thyroid tissue antigen due to destructive thyroiditis.

In summary, serial monitoring of thyroid tests and close surveillance is important to distinguish autoimmune thyroid disease induced by ICI therapy and Graves' disease. Baseline thyroid tests are recommended prior to initiation of ICI therapy [5]. ICI-induced thyrotoxicosis is usually managed symptomatically with beta-blockers and transient thyroiditis evolves into hypothyroidism. Similarly, a patient like ours with pre-existing mild Graves' disease can be treated symptomatically without ATD, as there is a possibility of destructive thyroiditis and subsequent hypothyroidism. Close monitoring is advised for use of ATD in cancer patients who may be on concurrent chemotherapy and ICI therapy which puts them at additional risk for bone marrow suppression and hepatic toxicity. ATD use could potentially be avoided in these patients unless they develop moderate to severe thyrotoxicosis and uncontrolled symptoms despite symptomatic treatment. Thyroid antibodies like TSH receptor antibodies (TRAb) or TSI are useful to distinguish Graves' disease vs thyroiditis but can be negative [10]. A radioactive scan can further differentiate thyroiditis present with decreased uptake, like our patient, vs increased uptake in Graves' disease (Fig. 1).

This is a unique case of a patient with Graves' disease who went into a prolonged remission or potentially cured because of immune-related adverse events from use of immune checkpoint inhibitor therapy.

## Learning Points

- Immune checkpoint inhibitor (ICI) therapy is commonly and frequently used in many malignancies
- Endocrine immune-related adverse events (irAEs) are common and the thyroid is the most common organ involved
- Baseline and serial monitoring of thyroid function tests is very important

## Data Availability

Not applicable to this article as no datasets were generated or analyzed during the current study.

## Abbreviations

ATD: antithyroid drug
CTLA-4: cytotoxic T-lymphocyte-associated protein 4
ICI: immune checkpoint inhibitor
irAE: immune-related adverse events
PD-1: programmed cell death protein 1
PD-L1: programmed cell death ligand 1
TPO: thyroid peroxidase
TSH: thyrotropin (thyroid-stimulating hormone)
TSI: thyroid-stimulating immunoglobulin
